# 648. A Tiered Framework for Burn Surgery Anesthesia

**DOI:** 10.1093/jbcr/irag033.485

**Published:** 2026-04-06

**Authors:** Cherry Song, Lane L Palmisano, Abraham Houng

**Affiliations:** The Burn Center at Cooperman Barnabas, Livingston, NJ; St. George's University School of Medicine, Livingston, NJ; The Burn Center at Cooperman Barnabas, Livingston, NJ

## Abstract

**Introduction:**

Although burn care presents unique challenges for anesthesia management, education in this area remains limited, as burns are frequently categorized within the broader scope of trauma in training curricula. As a result, the depth of exposure to burn-specific management in clinical practice is often inconsistent, leading to variability in provider experience. This highlights the need for improved communication with the anesthesia team to ensure both patient safety and quality of care. One potential strategy to address these gaps is the implementation of a tiered system that stratifies patients according to acuity. Such a framework could better align case complexity with anesthesia expertise, standardize clinical decision-making, and provide more consistent educational opportunities for trainees.

**Methods:**

A single-center retrospective study was conducted, reviewing burn surgical cases over a one-year period from September 1, 2024, to August 31, 2025. Patients were classified into three categories: Green, comprising stable patients such as step-down or outpatient cases that do not require a specialized anesthesiologist; Yellow, encompassing patients with significant burns and potential for blood loss, for whom an experienced anesthesiologist is preferred; and Red, including critically ill patients, often mechanically ventilated, with extensive burns and high anticipated blood loss, necessitating management by an experience anesthesiologist.

**Results:**

A total of 132 burn cases, including both adult and pediatric patients, were categorized using the tiered system. There were 72 green cases, 30 yellow cases, and 30 red cases.

**Conclusions:**

Our single-center implementation of a tiered burn patient acuity system demonstrates that stratifying cases by complexity is feasible and can guide anesthesia planning effectively. During the one-year review, case distribution across the green, yellow, and red tiers reflected a balanced mix of patient acuity, suggesting that the system captures meaningful clinical differences. While these findings are limited by a single-center design and retrospective review, the results support improved standardization of perioperative management, targeted allocation of anesthesia expertise, and opportunities for focused trainee education.

**Applicability of Research to Practice:**

Highly applicable for anesthesia inclusion and patient safety, with potential to extend to metrics such as blood and fluid given in the OR.

**Funding for the study:**

N/A.

